# Transcriptomic Analysis Reveals PACS1 as a Potential Shared Candidate Biomarker for Apical Periodontitis and Osteoporosis

**DOI:** 10.3390/ijms27156646

**Published:** 2026-07-25

**Authors:** Saixuan Wu, Sen Wang, Huicong Bai, Jingyantong Zhang, Zhen Zhang, Ming Dong, Weidong Niu

**Affiliations:** School and Hospital of Stomatology, Dalian Medical University, Dalian 116044, China; wusx@dmu.edu.cn (S.W.); wangs29@dmu.edu.cn (S.W.); baihc@dmu.edu.cn (H.B.); zhangjingyantong@outlook.com (J.Z.); zhangz011210@163.com (Z.Z.)

**Keywords:** apical periodontitis, osteoporosis, PACS1, immune infiltration, osteoblast differentiation

## Abstract

Apical periodontitis (AP), a localized inflammatory bone disease, and osteoporosis (OP), a systemic metabolic bone disorder, are both characterized by chronic inflammation and dysregulated bone metabolism, yet the molecular mechanisms underlying their association remain unclear. This study aimed to identify shared biomarkers and pathogenic pathways linking AP and OP. Gene expression datasets for AP and OP were obtained from the Gene Expression Omnibus database, and shared differentially expressed genes were identified and analyzed using functional enrichment, Gene set enrichment analysis (GSEA), Gene set variation analysis GSVA, and single-sample gene set enrichment analysis (ssGSEA)-based immune infiltration approaches. A random forest model was applied to prioritize candidate genes, followed by validation using single-cell RNA sequencing datasets, clinical samples, and in vitro assays. Forty-three shared differentially expressed genes were identified, among which *FAM87B*, *ASCL2*, and *PACS1* were prioritized as candidate genes. *PACS1* showed consistent upregulation in both AP and OP and was further validated in clinical samples. Integrated analyses suggested that shared molecular alterations were associated with NF-κB signaling and immune-metabolic dysregulation, while single-cell analysis supported *PACS1* expression in cell subsets involved in inflammatory bone remodeling. Functionally, *PACS1* knockdown promoted osteoblast differentiation, accompanied by increased ALP, RUNX2, and OPN expression. These findings suggest that *PACS1* may serve as a shared candidate biomarker linking AP and OP and provide insight into immune-bone crosstalk in inflammatory bone loss.

## 1. Introduction

Apical periodontitis (AP) is an inflammatory disease of the periapical region resulting from pulp necrosis [1]. Its key pathological features include granuloma formation and local alveolar bone destruction [2]. Although lesions are confined to the oral cavity, a growing body of evidence has yielded a key observation [3]. Lipopolysaccharide (LPS) and various proinflammatory cytokines released from AP lesions can enter the systemic circulation. These factors are associated with multiple systemic disorders, including cardiovascular diseases, diabetes mellitus, pregnancy complications, osteoporosis (OP), and autoimmune diseases [4]. OP is a systemic skeletal disorder, with imbalanced bone metabolism as its core pathological mechanism. Its essence lies in the persistent dominance of bone resorption over bone formation [5]. This metabolic imbalance ultimately leads to reduced bone mass, bone tissue degeneration, and impaired bone microstructure [6]. The development of OP is closely linked to decreased estrogen levels [7]. For this reason, it shows an exceptionally high prevalence among postmenopausal women. It is thus recognized as a typical characteristic disease of this population [8].

Multiple studies have provided evidence to support the clinical association between AP and OP. OP patients exhibit a significantly higher prevalence of AP lesions [9]. A survey study demonstrated that the prevalence of AP reaches 1.78% in OP patients, which is markedly higher than the 0.52% reported in the general population [10]. Further research has identified a positive correlation between the volume of periapical lesions and the degree of bone mineral density reduction [11]. In addition, animal model experiments have confirmed that rats with OP induced by ovariectomy show more pronounced periapical bone resorption in AP lesions [12,13]. Collectively, these clinical and experimental lines of evidence suggest that chronic local inflammation caused by AP may affect systemic bone metabolic balance through systemic signal transduction. Conversely, the systemic osteoporotic state may impair the healing capacity of periapical lesion tissues. This forms a bidirectional relationship between local inflammation and systemic bone metabolic disorder [14]. Mechanistically, the bidirectional relationship between AP and OP is likely mediated by shared inflammatory signaling networks, particularly the activation of the NF-κB pathway and the ubiquitin-proteasome system. Previous studies have established that these pathways are critical regulators of osteoclast differentiation in periapical inflammation and are also central to bone loss in postmenopausal osteoporosis [15,16,17,18]. Therefore, we hypothesized that although the initiating insults differ, AP and OP may converge on common functional modules involving immune-metabolic dysregulation and osteoclastogenesis.

Both AP and OP are globally prevalent diseases [19,20]. They share similar and synergistic pathological processes in terms of imbalanced bone metabolism and chronic inflammation [21]. Elucidating their interrelationship in depth is of great significance for developing therapeutic strategies that simultaneously improve oral and systemic health.

**Figure 1:**
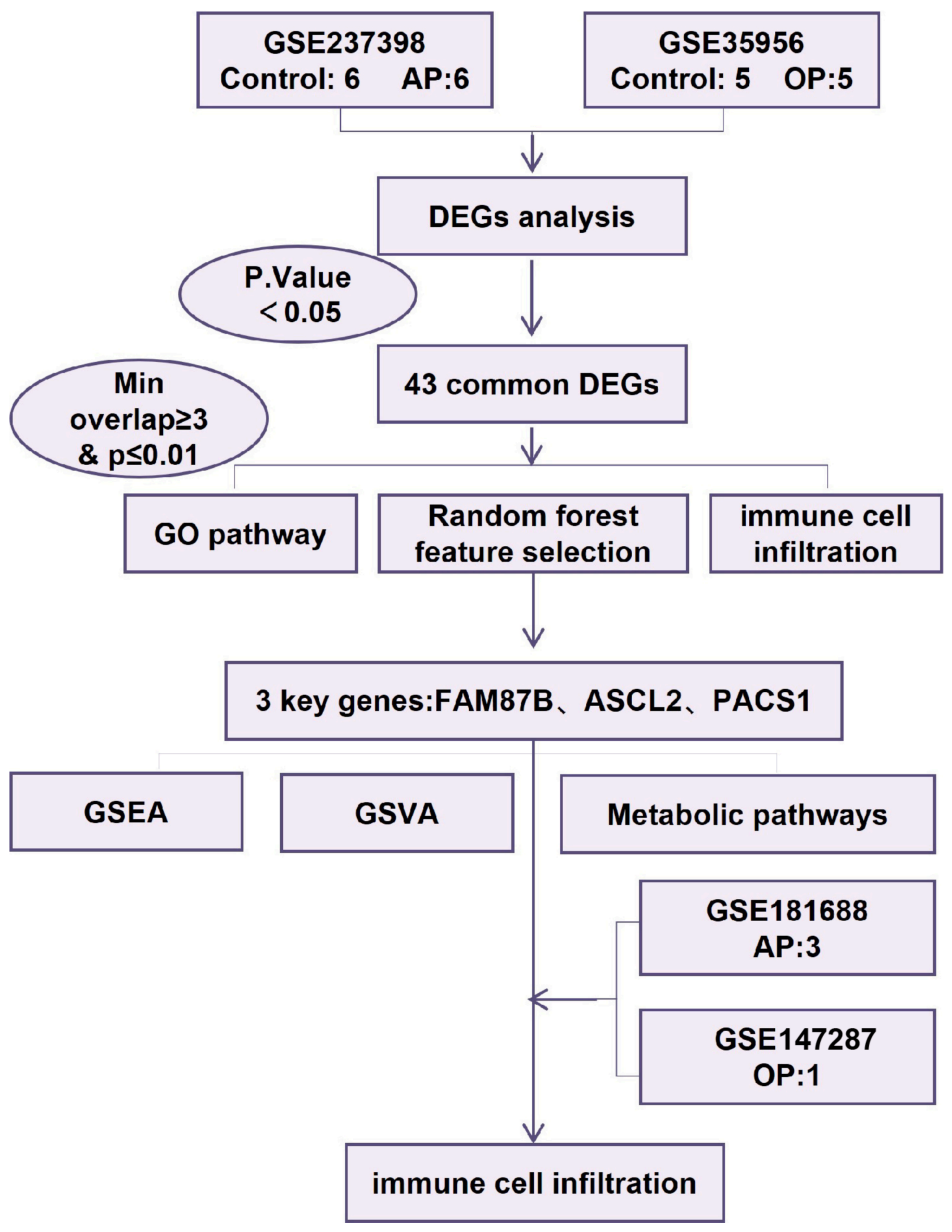
Research flowchart. Apical periodontitis (AP); Osteoporosis (OP); Differentially Expressed Genes (DGEs); Gene Ontology (GO); Gene Set Enrichment Analysis (GSEA); Gene Set Variation Analysis (GSVA).

In this study, we applied RNA transcriptomics to identify differentially expressed genes (DEGs) related to AP and OP and to analyze their association with immune cell infiltration. Furthermore, we performed single-cell RNA sequencing (scRNA-seq) to validate the expression profiles of these core candidate genes. We further elucidated the molecular mechanisms underlying the role of these genes in AP and OP pathogenesis. These findings provide novel insights into diagnostic and therapeutic approaches. The workflow of the present study is illustrated in Figure 1.

## 2. Results

### 2.1. Transcriptomic Integration Reveals Shared Molecular Signatures Between AP and OP

**Figure 2:**
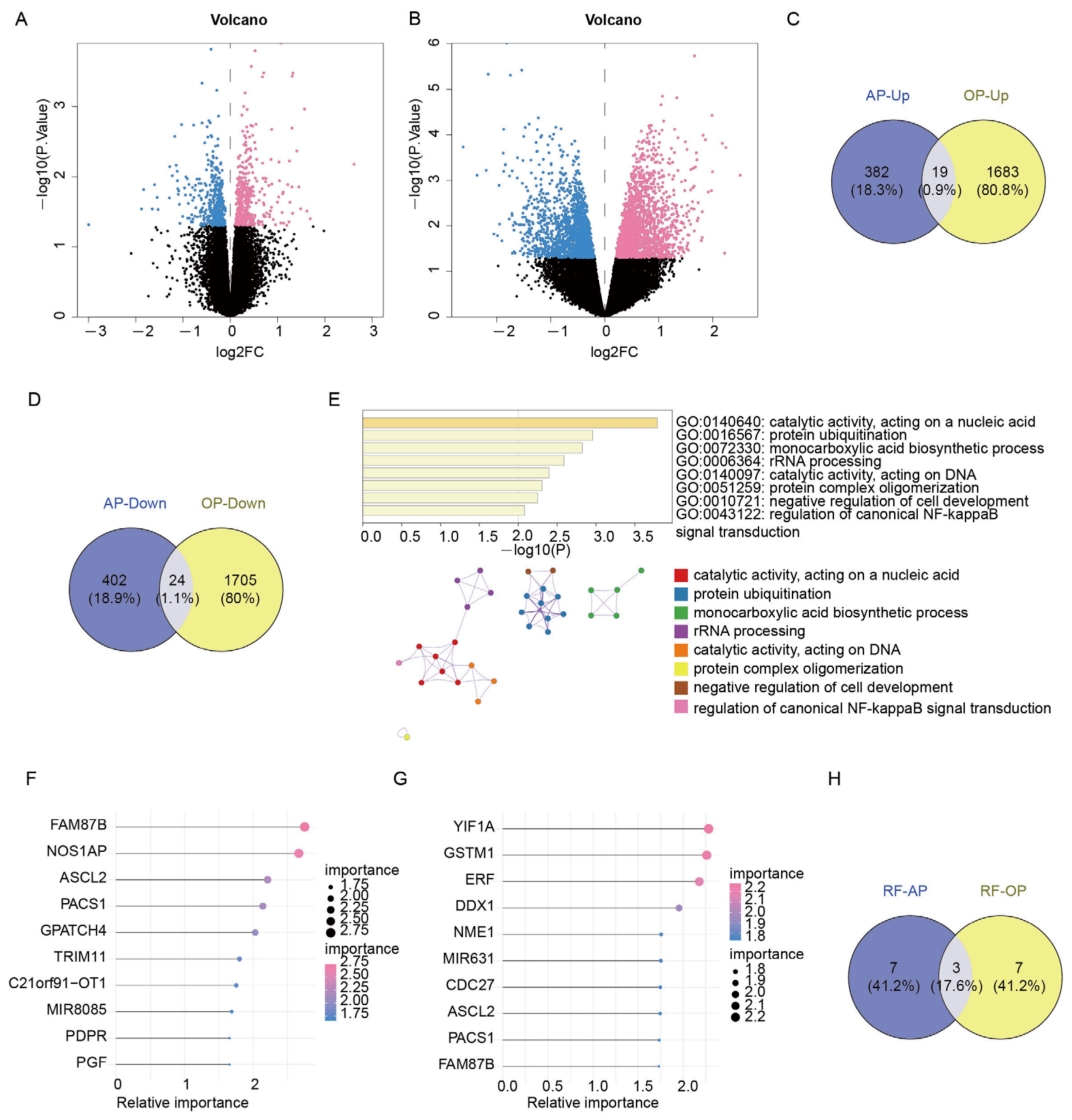
DEG analysis, functional enrichment and key gene screening in AP and OP. (**A**) Volcano plot of DEGs in AP (GSE237398, limma, *p* < 0.05). (**B**) Volcano plot of DEGs in OP (GSE35956, limma, *p* < 0.05). Pink dots: significantly up-regulated genes; blue dots: significantly down-regulated genes; black dots: non-significantly changed genes. (**C**) Venn diagram: 19 co-upregulated DEGs between AP and OP. (**D**) Venn diagram: 24 co-downregulated DEGs between AP and OP. (**E**) Metascape enrichment analysis of the 43 overlapping DEGs. The upper bar plot presents the significance of the enriched GO terms, measured by −log10 (*p* value). The lower gene-concept network depicts associations between genes and enriched GO terms: nodes represent genes mapped to the enriched terms, with node colors corresponding to the GO terms shown in the legend. Edges indicate shared term membership, and the network comprises four functional modules based on gene-term connectivity. (**F**) Top 10 AP characteristic genes. (**G**) Top 10 OP characteristic genes. (**H**) Venn diagram: 3 overlapping key genes.

According to the methods section, we performed differential expression analysis on datasets GSE237398 and GSE35956. A total of 827 DEGs were identified in GSE237398, including 401 upregulated and 426 down-regulated genes (Figure 2A). In GSE35956, 3431 DEGs were obtained, comprising 1702 upregulated and 1729 down-regulated genes (Figure 2B). A total of 43 DEGs identified from the differential expression analysis were shared between the two datasets, including 19 co-upregulated and 24 co-downregulated genes (Figure 2C,D). Although the overlap at the individual gene level was limited, these shared DEGs suggest partial convergence between AP and OP at the molecular level. Further functional enrichment analysis using Metascape showed that these overlapping genes were mainly associated with catalytic activity acting on nucleic acid, protein ubiquitination, monocarboxylic acid biosynthetic process, and regulation of canonical NF-κB signaling (Figure 2E), indicating convergence at the pathway and process levels rather than extensive gene-level overlap. The accompanying gene-concept network illustrates the associations between the overlapping genes and the enriched GO terms, with nodes representing genes mapped to the enriched terms and node colors corresponding to the indicated functional terms. The network was organized into four functional modules based on shared gene-term connectivity. Collectively, these results suggest that the shared molecular alterations between AP and OP converge on multiple interconnected biological processes rather than isolated pathways.

### 2.2. Identification of Key Molecular Signatures Linking AP and OP

To identify key genes associated with AP and OP, the random forest algorithm was applied to the previously obtained intersection genes. The top 10 characteristic genes for AP were selected (Figure 2F), and the top 10 characteristic genes for OP were identified using the same approach (Figure 2G). Three common genes were obtained from the intersection of these two gene sets (Figure 2H): *FAM87B*, *ASCL2*, and *PACS1*, which were defined as the key candidate genes for subsequent analyses. The ROC curves of the random forest classification models for AP and OP are shown in Supplementary Figure S1, with AUC values > 0.85 for both models, indicating reliable predictive performance of the screened gene signature.

### 2.3. Shared Immune Characteristics Emerge from AP and OP Molecular Signatures

**Figure 3:**
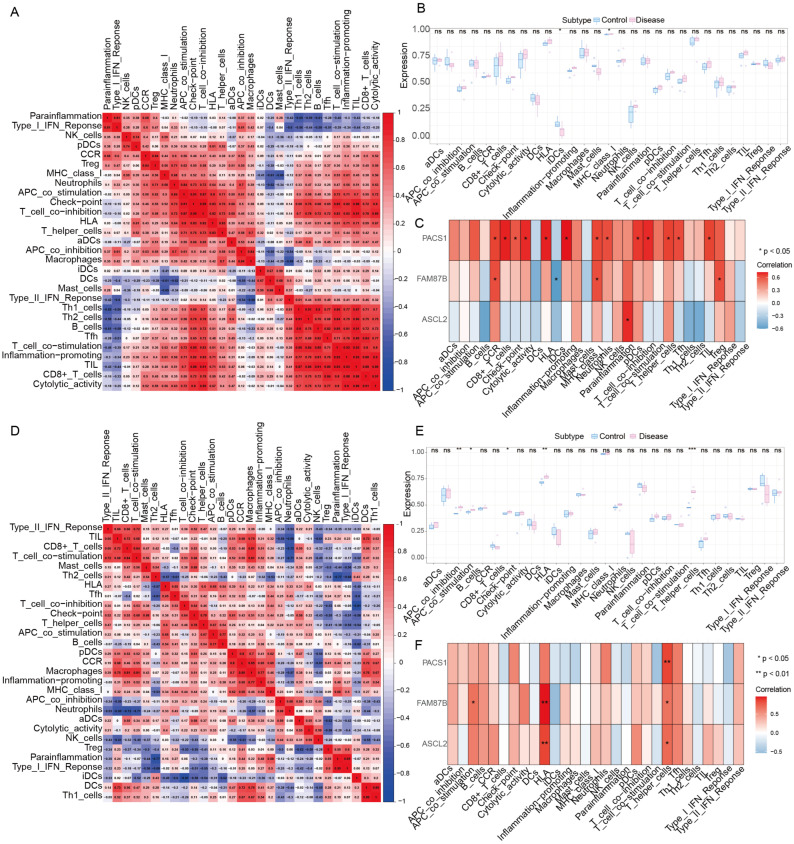
Immune cell correlation, infiltration level comparison, and key gene association analysis in AP and OP. (**A**) Heatmap showing immune cell correlation and infiltration level distribution in the AP dataset. (**B**) Boxplot comparing MHC class I and iDCs infiltration levels between AP disease and control groups. (**C**) Heatmap showing the correlation between key genes (*FAM87B*, *ASCL2*, *PACS1*) and immune cell-related indicators in the AP dataset. (**D**) Heatmap showing immune cell correlation and infiltration level distribution in the OP dataset. (**E**) Boxplot comparing APC co-stimulation, B cells, checkpoint, HLA, and T helper cells infiltration levels between OP disease and control groups. (**F**) Heatmap showing the correlation between key genes (*FAM87B*, *ASCL2*, *PACS1*) and immune cell-related indicators in the OP dataset. * *p* < 0.05, ** *p* < 0.01, *** *p* < 0.001, ns: not significant.

The immune microenvironment is primarily composed of infiltrating immune cells and soluble mediators, such as cytokines and chemokines [22]. To characterize the inferred immune microenvironment, we performed single-sample gene set enrichment analysis (ssGSEA)-based inference of immune-related signatures in the AP and OP datasets. In the AP dataset, the disease group showed a significantly higher level of MHC class I activity and a significantly lower level of immature dendritic cells (iDCs) than the control group (Figure 3A,B). Correlation analysis revealed distinct immune association patterns for the three key genes (Figure 3C). *FAM87B* showed positive correlations with CCR, MHC class I, and Treg, but a negative correlation with iDCs. *ASCL2* was positively associated with parainflammation. *PACS1* exhibited widespread positive correlations with multiple immune components, including CCR, CD8^+^T cells, checkpoint molecules, cytolytic activity, HLA, inflammatory mediators, MHC class I, neutrophils, pDCs, T-cell co-inhibition, T helper cells, Tfh, and TIL.

In the OP dataset, we further explored the inferred immune infiltration profile and its correlation with immune cell populations using multiple visualization strategies (Figure 3D). The disease group exhibited significantly elevated levels of APC co-stimulation, B cells, checkpoint molecules, HLA, and T helper cells relative to the control group (Figure 3E). Correlation analysis between key genes and immune profiles demonstrated that *FAM87B* was markedly positively associated with APC co-stimulation, HLA, and T helper cells. *ASCL2* showed a significantly positive correlation with HLA and T helper cells, while *PACS1* was positively correlated with T helper cells (Figure 3F).

### 2.4. Functional Pathways Associated with AP–OP Key Genes Reveal Convergent and Disease-Specific Regulatory Programs

**Figure 4:**
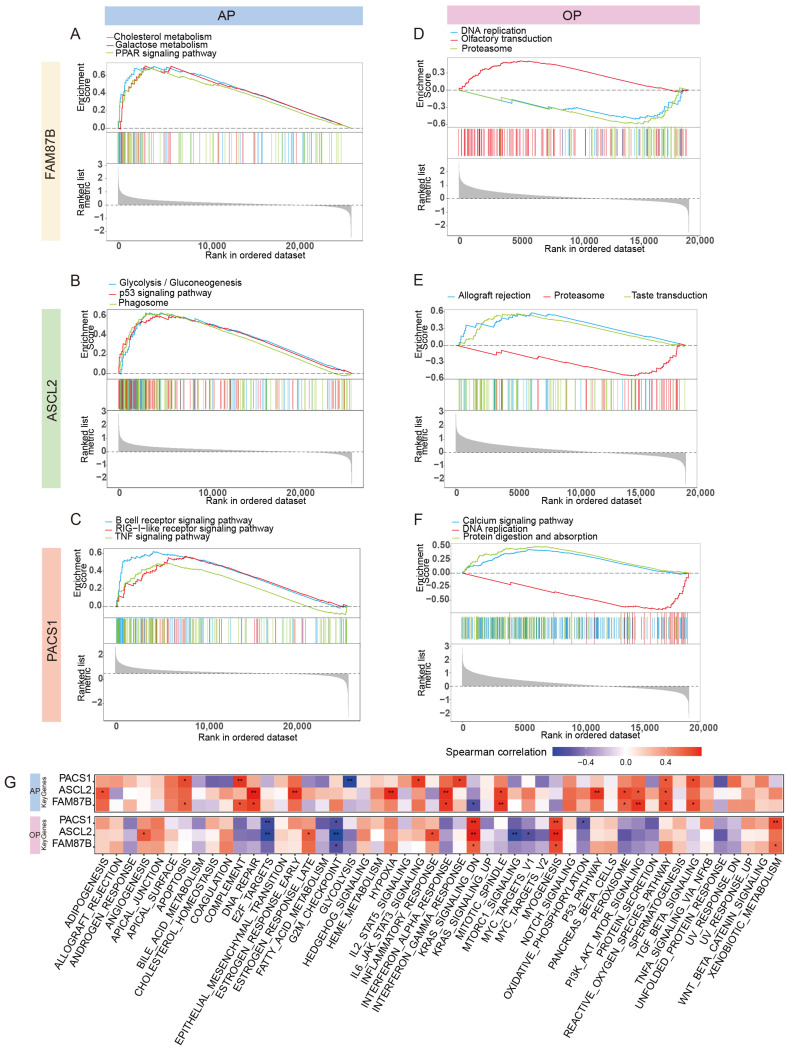
GSEA and GSVA enrichment analysis of key genes in AP and OP. (**A**–**C**) GSEA of key genes in AP: (**A**) *FAM87B*, (**B**) *ASCL2*, (**C**) *PACS1*. (**D**–**F**) GSEA of key genes in OP: (**D**) *FAM87B*, (**E**) *ASCL2*, (**F**) *PACS1*. (**G**) GSVA of key genes in AP and OP. * *p* < 0.05, ** *p* < 0.01.

To explore the potential molecular mechanisms of key genes in disease progression, GSEA was performed. In AP, *FAM87B* was enriched in PPAR signaling, cholesterol metabolism, and galactose metabolism pathways (Figure 4A). *ASCL2* showed enrichment in phagosome, glycolysis/gluconeogenesis, and p53 signaling pathways (Figure 4B). *PACS1* was associated with B-cell receptor signaling, RIG-I-like receptor signaling, and TNF signaling pathways (Figure 4C).

GSEA of the OP dataset revealed distinct pathway enrichments for each key gene. *FAM87B* was enriched in proteasome, DNA replication, and olfactory transduction pathways (Figure 4D). *ASCL2* showed enrichment in proteasome, taste transduction, and allograft rejection pathways (Figure 4E). *PACS1* was primarily associated with DNA replication, calcium signaling, and protein digestion and absorption pathways (Figure 4F).

GSVA was performed to investigate pathway-level variations associated with key genes, complementing the group-level pathway enrichment identified by GSEA. In AP, representative pathway activity patterns associated with *FAM87B* and *ASCL2* included INTERFERON_ALPHA_RESPONSE and PI3K_AKT_MTOR_SIGNALING (Figure 4G), while those associated with *PACS1* included INTERFERON_GAMMA_RESPONSE and COMPLEMENT pathways. In OP, representative pathway activity patterns associated with *FAM87B*, *ASCL2*, and *PACS1* included KRAS_SIGNALING_DN and MYOGENESIS pathways. These results show that the key genes were enriched in disease-related pathways.

### 2.5. AP and OP Molecular Signatures Are Associated with Metabolic Remodeling

**Figure 5:**
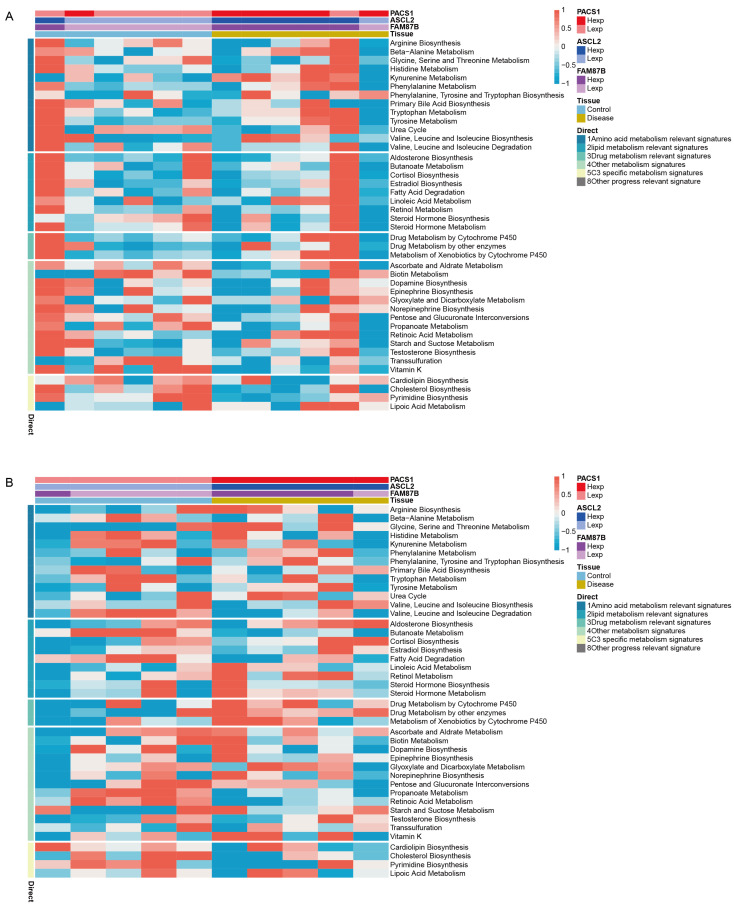
Correlation between key genes and metabolic pathways in AP and OP. (**A**) ssGSEA heatmap showing the positive correlation between key genes (*PACS1*, *ASCL2*, *FAM87B*) and metabolic pathways in AP dataset. (**B**) ssGSEA heatmap showing the positive correlation between key genes (*PACS1*, *ASCL2*, *FAM87B*) and metabolic pathways in OP dataset.

To investigate the association between key genes and metabolic pathways, predefined pathway-related gene sets were mapped to the transcriptomic profiles of AP and OP samples, and ssGSEA was performed to quantify the corresponding pathway enrichment scores as indirect measures of pathway activity in AP and OP, with results visualized as heatmaps (Figure 5A,B). In AP, *PACS1*, *ASCL2*, and *FAM87B* showed positive correlations with the kynurenine metabolism pathway. In OP, these three genes were positively correlated with glycine, serine, and threonine metabolism pathways. All statistical verification results are listed in Supplementary Table S3, and Propanoate Metabolism was recognized as a common metabolic pathway shared by the two disease groups.

### 2.6. Single-Cell Transcriptomic Profiling Reveals the Cellular Distribution of AP and OP-Associated Key Genes

After quality control and doublet removal, 21,375 cells were retained for downstream analysis. The filtered datasets were visualized using violin and scatter plots (Figure S2A,B). A total of 2000 highly variable genes were identified, followed by normalization, PCA, and Harmony-based batch correction (Figure S2C–E).

Following quality control and doublet removal, 7044 valid OP cells were retained for subsequent analysis. The data distribution and quality after filtering were visualized using violin and scatter plots (Figure S3A,B). A total of 2000 highly variable genes were identified, and the data were then normalized, scaled, and subjected to PCA and Harmony-based batch correction (Figure S3C–E).

**Figure 6:**
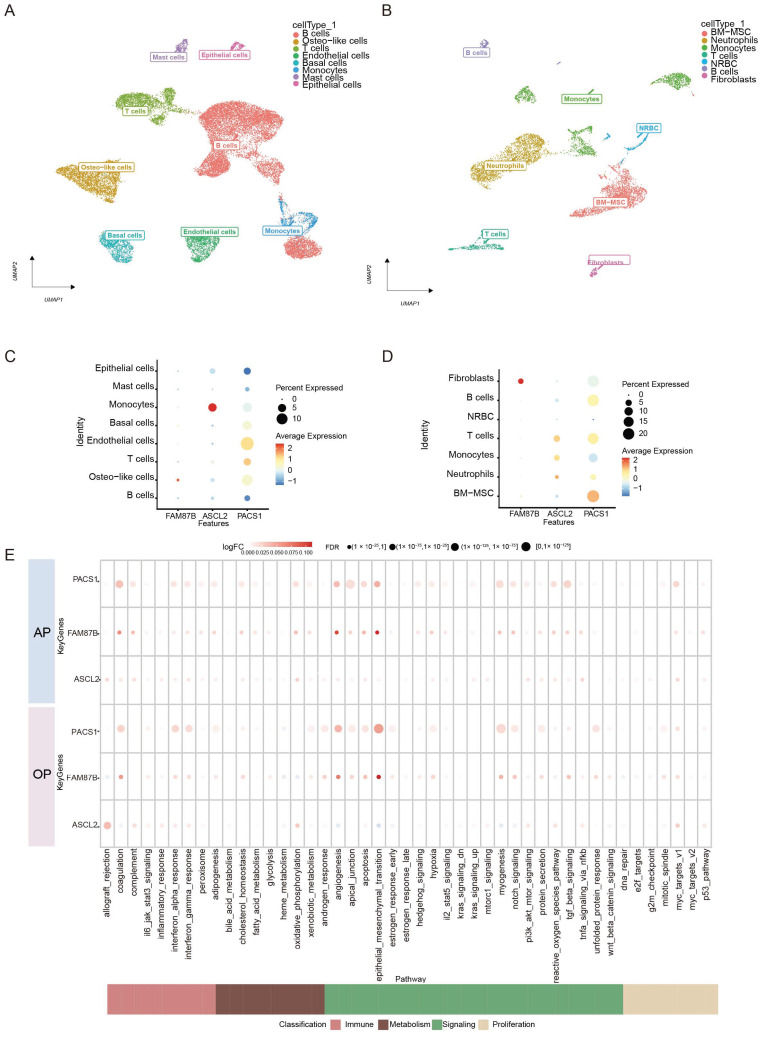
UMAP annotation, key gene expression and immune metabolic pathway analysis of AP and OP scRNA-seq datasets. (**A**) UMAP dimensionality reduction and annotation of 21,375 cells from the AP scRNA-seq dataset into 8 cell categories via known markers. (**B**) UMAP dimensionality reduction and annotation of 7044 cells from the OP scRNA-seq dataset into 7 cell types via known markers. (**C**) Dot plot showing the expression levels of the three key genes across different cell populations in AP patients. (**D**) Dot plot showing the expression levels of the three key genes across different cell populations in OP patients. (**E**) Dot plot of key gene expression in immune metabolic pathways in AP and OP samples.

In both AP and OP single-cell datasets, 12 cell clusters were identified and further annotated into distinct cell subtypes. For the AP dataset, the 12 clusters were finally classified into 8 cell types, including B cells, osteo-like cells, T cells, endothelial cells, basal cells, monocytes, mast cells, and epithelial cells (Figure 6A). The expression profiles of three core key genes across these annotated cell types were characterized (Figure 6C). For the OP dataset, 12 subpopulations were annotated into 7 cell types, including BM-MSC, neutrophils, monocytes, T cells, NRBC, B cells, and fibroblasts (Figure 6B), and the characteristic gene expression patterns of each cell subtype were presented (Figure 6D).

### 2.7. Key Genes Exhibit Distinct Immune-Metabolic Activity Patterns at Single-Cell Resolution

Disease-related genes were selected from GeneCards based on their relevance scores. AUCell was used to score immune metabolism-related pathway activity at the single-cell level [23]. Bubble plots showed the associations between key genes and pathway activity (Figure 6E). *FAM87B*, *ASCL2*, and *PACS1* were associated with relatively high activity in oxidative phosphorylation and epithelial-mesenchymal transition (EMT) pathways in AP.

Disease-related genes were selected from GeneCards based on their relevance scores. AUCell was used to score immune metabolism-related pathway activity at the single-cell level. Bubble plots showed the associations between key genes and pathway activity (Figure 6E). *FAM87B* and *PACS1* were associated with relatively high activity in EMT-related pathways, while *ASCL2* was associated with relatively high activity in the allograft rejection pathway in OP.

**Figure 7:**
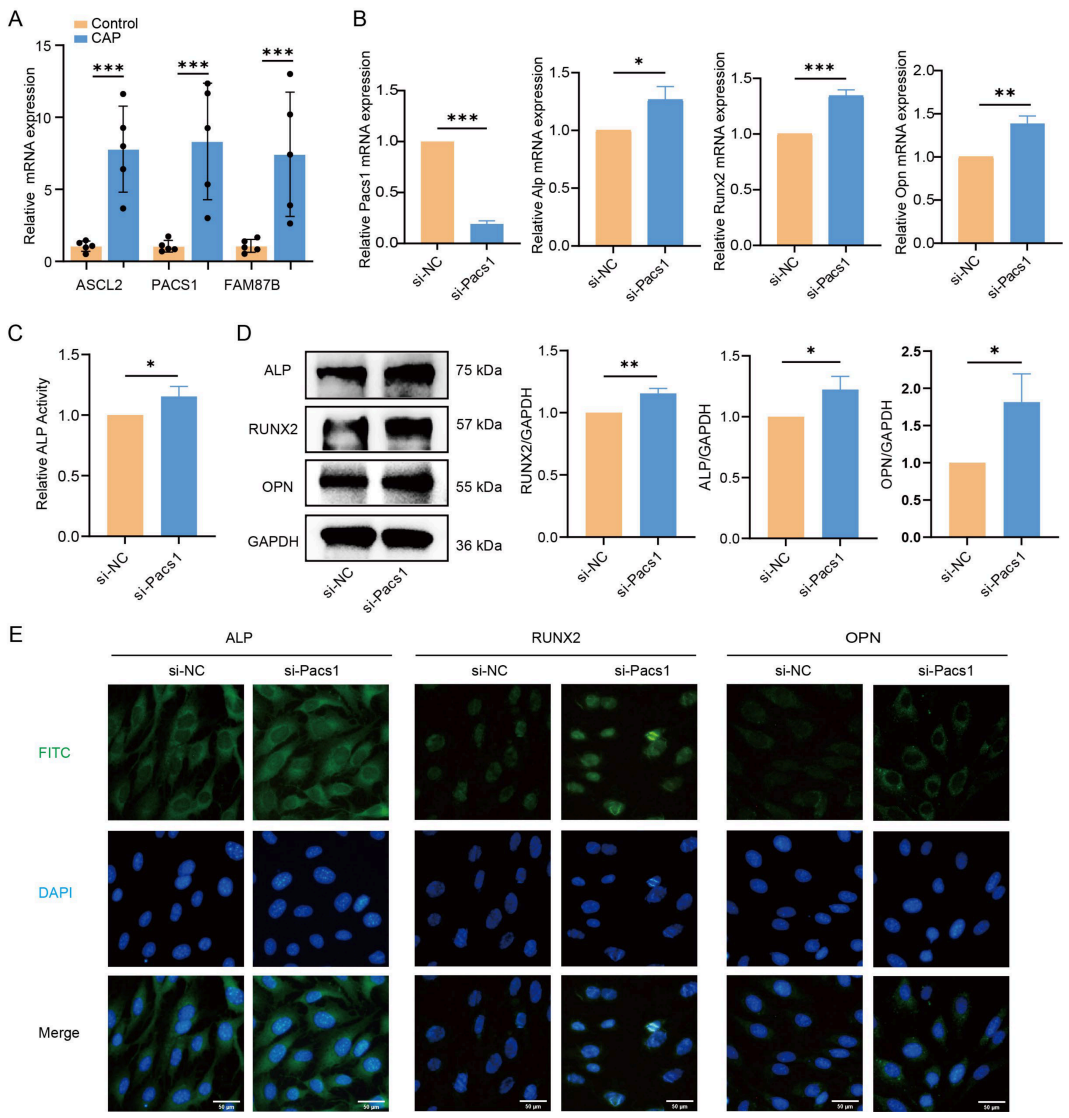
PACS1 upregulation in comorbid samples and its transient knockdown enhances osteoblast differentiation. (**A**) qRT-PCR analysis of *PACS1* expression in clinical samples from 5 patients with comorbid AP and OP. (**B**) qRT-PCR detection of *Alp*, *Runx2* and *Opn* mRNA levels in osteoblasts. (**C**) ALP activity assay. (**D**) Western blot verification of ALP, RUNX2 and OPN protein levels. (**E**) IF staining showing the localization and expression of ALP, RUNX2 and OPN in osteoblasts. Scale bar = 50 μm. * *p* < 0.05, ** *p* < 0.01, *** *p* < 0.001.

Among the three candidate genes identified by random forest analysis, *PACS1* was prioritized for subsequent experimental validation because of its consistent association with immune-related features and its biological relevance. We collected clinical samples from 5 patients with comorbid AP and OP. qRT-PCR results demonstrated that the expression of *PACS1* was significantly upregulated in these samples compared with the control group (*p* < 0.05) (Figure 7A). Subsequently, we transiently knocked down *PACS1* expression in osteoblasts via transfection; qRT-PCR analysis showed that the mRNA expression levels of alkaline phosphatase (ALP), runt-related transcription factor 2 (RUNX2), and osteopontin (OPN) were all upregulated (*p* < 0.05) (Figure 7B). Compared with the negative control (NC) group, ALP activity was significantly increased in the si-PACS1 group (*p* < 0.05) (Figure 7C). Western blot results were consistent with those of qRT-PCR (*p* < 0.05) (Figure 7D). IF staining results revealed that ALP was expressed in both the cytoplasm and nucleus, with enhanced fluorescence signals observed in the si-PACS1 group compared with the NC group. RUNX2 was predominantly expressed in the nucleus, and its fluorescence signals were also increased in the si-PACS1 group relative to the NC group. OPN was mainly localized in the cytoplasm, and the si-PACS1 group exhibited stronger fluorescence signals than the NC group (Figure 7E). These results indicate that PACS1 knockdown was associated with increased expression of osteoblast differentiation markers.

## 3. Discussion

Bones are increasingly recognized as metabolically and immunologically active organs that maintain mineral storage, electrolyte homeostasis, and systemic energy metabolism [24]. The bone microenvironment is shaped by complex interactions among osteoblasts, osteoclasts, stromal cells, and immune cells, and disruption of this immune-bone network contributes to inflammatory bone loss [25,26,27]. During periapical inflammatory processes, lymphocytes and non-immune cells, including osteoblast-like cells, stromal cells, and epithelial cells, are intermingled within the lesions [28]. In the present study, immune-related analyses indicated that both AP and OP were characterized by immune activation, although their immune signatures differed between the two conditions. In AP lesions, the upregulation of MHC class I-related signatures, together with altered iDC abundance, suggested a disturbance of antigen presentation-associated immune activity, which may contribute to persistent local inflammation. Immune cell infiltration also plays a critical role in the initiation and progression of OP. The balance among Treg cells, T helper 17 cells, Bregs, and macrophages is essential for maintaining osteoblast-osteoclast homeostasis, thereby modulating the development of OP [29,30,31]. In our analysis of OP, activation of APC co-stimulatory pathways and increased HLA-related signatures indicated enhanced immune responses. Increased B-cell and T helper cell-related signatures may further aggravate inflammatory activation, thereby disturbing osteoblast-osteoclast homeostasis and contributing to bone metabolic imbalance.

PACS1 is an intracellular sorting protein that recognizes acidic cluster motifs and mediates protein sorting and localization to the trans-Golgi network [32]. It also regulates protein trafficking in secretory and endocytic pathways [33] and has been implicated in several biological processes, including viral immune escape, neural development, and nucleocytoplasmic transport [34,35,36]. Given that antigen presentation depends on coordinated intracellular trafficking, vesicular sorting, and membrane localization of MHC/HLA molecules, the association between PACS1 and immune-related signatures observed in this study suggests that PACS1 may participate in antigen presentation-related or immune regulatory processes in AP and OP.

Correlation analysis further showed that PACS1 was positively associated with multiple immune-related features in AP, including CCR, CD8^+^T cells, immune checkpoints, cytolytic activity, HLA, inflammatory promotion, MHC class I, neutrophils, pDCs, T-cell co-inhibition, T helper cells, Tfh cells, and TILs. In contrast, PACS1 showed a significant positive correlation only with T helper cells in OP. These results suggest that PACS1 may be more broadly associated with immune regulation in AP, whereas its immune-related role in OP may be mainly linked to T helper cell-associated responses. The consistent association between PACS1 and T helper cells in both diseases is noteworthy, as activated T helper cells can release proinflammatory cytokines, including IL-1β, TNF-α, and IL-6, which are important mediators of inflammatory bone destruction [37]. In AP, these inflammatory mediators may contribute to periodontal ligament damage, alveolar bone resorption, and persistent local inflammation. In OP, similar inflammatory changes may promote osteoclast differentiation and suppress osteoblast function, partly through the RANKL/RANK pathway, thereby shifting bone remodeling toward bone resorption.

We further validated PACS1 expression in clinical samples and found that PACS1 was elevated in patients with comorbid AP and OP. Functionally, PACS1 knockdown in osteoblasts promoted osteogenic differentiation, consistent with the findings reported by Zhu et al. [38]. These findings suggest that elevated PACS1 may be associated not only with immune-related alterations but also with impaired osteogenic potential. The consistent upregulation of PACS1 across AP and OP, together with its functional effect on osteoblast differentiation, supports its role as a shared molecular link between the two diseases and a candidate target for further therapeutic investigation. Previous studies have also suggested that PACS1 may regulate inflammatory responses through PAR1/PAR2-related mechanisms, although whether this pathway contributes to AP- or OP-associated bone remodeling remains to be further investigated [35].

Notably, PACS1 was also associated with increased activity of EMT-related gene signatures in both diseases. EMT is a biological process in which epithelial cells acquire mesenchymal characteristics and is closely related to cell migration, extracellular matrix remodeling, and inflammatory signaling [39,40]. Rather than necessarily indicating canonical epithelial-to-mesenchymal transition in all tissue contexts, this signature may reflect broader processes, including stromal activation, extracellular matrix remodeling, cell migration, and inflammation-associated microenvironmental remodeling. These findings suggest that PACS1 may be involved not only in immune dysregulation but also in remodeling of the inflammatory bone microenvironment during disease progression. However, the precise mechanisms underlying PACS1-mediated immune regulation and bone remodeling remain to be elucidated.

This study was based on publicly available GEO datasets; therefore, some potential confounding factors could not be fully assessed. In addition, the sample size of the discovery cohorts was modest, which may have affected the robustness and generalizability of the results. Although our findings provide a transcriptomic reference for AP and OP, they have not yet been validated in an independent transcriptomic dataset, and their reproducibility remains to be confirmed. Moreover, although PACS1 expression was validated at the clinical mRNA level, further spatial validation of immune cell infiltration using immunohistochemistry (IHC) in patient tissues will be necessary in future studies to confirm the precise localization of PACS1 and its interaction with specific immune cells. These limitations highlight the need for future studies with larger cohorts and external validation to further establish the clinical utility of our findings.

## 4. Materials and Methods

### 4.1. Data Download

Gene expression profiling data of apical periodontitis (AP, GSE237398) and osteoporosis (OP, GSE35956) were acquired from the Gene Expression Omnibus (GEO, https://www.ncbi.nlm.nih.gov/geo (accessed on 20 February 2026)). GSE237398 (annotated to GPL21290) included 12 expression profile samples with 6 cases in the control group and 6 in the disease group, while GSE35956 (annotated to GPL570) contained 10 samples with 5 controls and 5 disease cases. Single-cell RNA sequencing data of GSE181688 and GSE147287 were also retrieved from GEO, with 3 and 1 disease samples with complete single-cell expression profiles selected for subsequent single-cell analysis, respectively. Figure 1 shows the workflow chart of the present study. The detailed metadata available for each GEO dataset, including sample composition and reported demographic information, are summarized in Supplementary Tables S1 and S2.

### 4.2. Differential Expression Genes (DGEs) Analysis

DEGs in the expression profiles of AP and OP comorbidity were identified using the R package ‘limma’, with the screening condition of *p* value < 0.05, and we also drew volcano maps and heat maps of the DEGs.

### 4.3. Functional Enrichment Analysis

Functional annotation of differentially expressed genes was performed using the Metascape database (www.metascape.org) to fully explore the functional correlations of these genes. Gene Ontology (GO) pathway analysis was conducted on specific genes. Min overlap ≥ 3 and *p* ≤ 0.01 is considered statistically significant.

### 4.4. Random Forest Feature Selection

To further refine the selection of pivotal candidate genes, the random forest machine learning algorithm was applied to the expression profiles of AP and OP. The algorithm generates multiple decision trees via bootstrap sampling and random feature selection at each node. We fixed ntree = 100 and nPerm = 1000 for calculating the % IncMSE-based gene importance scores, and retained the top 10 genes ranked by importance for subsequent intersection analysis. In addition, independent random forest classification models were built for AP and OP, respectively, and ROC analysis was conducted to assess the predictive performance of the gene signature.

### 4.5. Analysis of Immune Cell Infiltration

The ssGSEA algorithm was employed to quantify the relative proportions of 29 immune cell types (including T cells, B cells, and NK cells) in the expression profiles of AP and OP [41]. The resulting ssGSEA scores were used as indirect indicators of immune cell infiltration. Correlation analysis between gene expression levels and immune cell infiltration was subsequently performed.

### 4.6. Gene Set Enrichment Analysis (GSEA)

Patients were stratified into high and low expression groups based on key gene expression levels. Gene set enrichment analysis (GSEA) was performed to identify differentially enriched signaling pathways between the two groups, using the annotated gene set (version 7.0) downloaded from the Molecular Signatures Database (MSigDB). Significantly enriched gene sets were defined by an adjusted *p*-value < 0.05.

### 4.7. Gene Set Variation Analysis (GSVA)

Gene set variation analysis (GSVA) was performed to evaluate pathway-level enrichment across samples [42]. Gene sets were downloaded from MSigDB. GSVA converts gene-level expression into comprehensive scores for each gene set, enabling assessment of potential biological functional changes between different samples.

### 4.8. Quality Control of Single-Cell Data

Single-cell expression profiles were read using the Seurat package. Cells were filtered based on UMI counts [43], number of expressed genes, and mitochondrial gene expression proportion. Specifically, cells with fewer than 200 captured genes were excluded in both AP and OP single-cell datasets. Quality control was performed using median absolute deviation (MAD); cells with values exceeding 3 MADs from the median were considered outliers and removed. DoubletFinder (V2.0.4) was subsequently applied to eliminate doublets, completing cell quality control.

### 4.9. Dimensionality Reduction, Clustering and Cell Annotation of Single-Cell Data

Single-cell data were normalized using the LogNormalize method (scale factor: 10,000) [44]. Cell cycle scores were calculated, and 2000 highly variable genes were identified for downstream analysis. Expression data were scaled to regress out mitochondrial/ribosomal content and cell cycle effects. Principal component analysis (PCA) was performed, followed by Harmony batch correction to remove batch effects among multiple samples. Uniform Manifold Approximation and Projection (UMAP) was subsequently used for nonlinear dimensionality reduction, and 12 cell clusters were identified in both the AP and OP datasets [45]. Cell types were annotated based on canonical marker genes using CellMarker and PanglaoDB databases, supplemented by SingleR.

### 4.10. Gene Acquisition and Single-Cell Pathway Activity Quantification

Disease-related genes were retrieved from the GeneCards database through keyword-based screening, and genes with the highest relevance scores were retained for subsequent analysis. The AUCell algorithm was applied to quantitatively score the activity of immune metabolism-related pathways at the single-cell resolution [23]. Bubble plots were generated to visualize the associations between key genes and immune metabolism-related pathway activity.

### 4.11. Study Population and Clinical Examinations

The study population consisted of 5 patients diagnosed with both AP and OP, and 5 control subjects who underwent orthodontic extraction of healthy teeth at the Stomatological Hospital of Dalian Medical University between June 2025 and December 2026. This study was approved by the Ethics Committee of the Affiliated Stomatological Hospital, School of Stomatology, Dalian Medical University (approval no. 2025004), and written informed consent was obtained from all participants. Detailed inclusion and exclusion criteria are provided in the Supplementary Materials.

### 4.12. Cell Culture

The murine pre-osteoblastic cell line MC3T3-E1 was purchased from Procell Life Science & Technology (Wuhan, China) and cultured in alpha-MEM supplemented with 10% fetal bovine serum (FBS) and 1% penicillin-streptomycin at 37 °C in a humidified atmosphere containing 5% CO_2_. Cells between passages 5 and 20 were used in this study.

### 4.13. Quantitative Real-Time Polymerase Chain Reaction (qRT-PCR)

The experiment included two groups: Negative Control group (NC group) (cells transfected with non-homologous negative control siRNA) and PACS1 Knockdown group (si-PACS1 group) (cells transfected with PACS1-targeted siRNA). 50 nM siRNA was transfected using Lipofectamine 3000 following the kit instructions (Thermo Fisher Scientific, Waltham, MA, USA). After 24 h of continuous culture, cells were collected for subsequent assays. Total RNA was extracted, with its concentration and purity determined subsequently. Complementary DNA was synthesized by reverse transcription. The mRNA expression levels of PACS1, ALP, RUNX2, and OPN were detected via qRT-PCR. GAPDH served as the internal reference gene, and the relative expression levels were calculated using the 2^(−ΔΔCt)^ method [46].

The primer sequences for human genes used for qRT-PCR analysis of clinical patient samples are listed below: *FAM87B* (Forward: 5′-TCTCAGGTTTGCACCTCGTC-3′; Reverse: 5′-ATCTAGATTGCTCAGGCCGC-3′), *ASCL2* (Forward: 5′-CTCGACCTATGAGCCTCAGC-3′; Reverse: 5′-ATCCTCTCCCCTCCGGAAAA-3′), *PACS1* (Forward: 5′-GCAGATGAACCTGTACGCCAC-3′; Reverse: 5′-CCCAAAGCCCACCTCATCTG-3′).

The primers used in the in vitro qRT-PCR assays in mouse MC3T3-E1 cells were as follows: *Pacs1* (Forward: 5′-GACTGGCTGGGTTACATGC-3′; Reverse: 5′-GCGACTGAATAGGTCTCTCCA-3′), *Alp* (Forward: 5′-TGAATCGGAACAACCTGACTGA-3′; Reverse: 5′-GAGCCTGCTTGGCCTTACC-3′), *Runx2* (Forward: 5′-CCTCTGGCCTTCCTCTCTCA-3′; Reverse: 5′-TAGGTAAAGGTGGCTGGGTAGTG-3′), *Opn* (Forward: 5′-GCTCAGCACCTGAATGTACC-3′; Reverse: 5′-CCTCGGCTCGATGGCTAGC-3′), *Gapdh* (Forward: 5′-GGCACAGTCAAGGCTGAGAATG-3′; Reverse: 5′-ATGGTGGTGAAGACGCCAGTA-3′).

The siRNA sequence targeting mouse Pacs1 (si-Pacs1) was: Sense: 5′-CGAACUAUCUUGGGCUAUAAATT-3′, Antisense: 5′-UUUAUAGCCCAAGAUAGUUCGTT-3′. Non-targeting scrambled siRNA (si-NC) was used as the negative control.

### 4.14. Western Blot

Protein lysates (20 μg/lane) were separated by SDS-PAGE, transferred to nitrocellulose membranes, and blocked with 5% non-fat milk (2 h). Membranes were incubated overnight at 4 °C with primary antibodies against OPN (Huabio, HA723082, 1:2000, Woburn, MA, USA), ALP (Huabio, ET1601-21, 1:5000), RUNX2 (Huabio, ET1612-47, 1:5000), and GAPDH (1:5000). After secondary antibody incubation (1:5000, 1 h, RT), bands were visualized by ECL and quantified using ImageJ 1.46r (National Institutes of Health, Bethesda, MD, USA).

### 4.15. Immunofluorescence Staining Assay

For immunofluorescence staining, samples were fixed in 4% paraformaldehyde (30 min, RT), permeabilized with 0.25% Triton X-100 (10 min), and subsequently blocked with goat serum for 1 h. The samples were then incubated overnight at 4 °C with primary antibodies against OPN (Huabio, HA723082, 1:2000), ALP (Huabio, ET1601-21, 1:1000), and RUNX2 (Huabio, ET1612-47, 1:2000). After washing, the samples were incubated with corresponding fluorescently labeled secondary antibodies (1 h, RT, dark). Nuclei were counterstained with DAPI. Images were captured using a fluorescence microscope.

### 4.16. Statistical Analysis

All statistical analyses were conducted using R language (version 4.3.0). All statistical tests were bilateral, and a *p* value < 0.05 was considered statistically significant.

## Data Availability

The original contributions presented in this study are included in the article/Supplementary Materials. Further inquiries can be directed to the corresponding authors.

## Supplementary Materials

The following supporting information can be downloaded at: https://www.mdpi.com/article/10.3390/ijms27156646/s1.

## Abbreviations

AP	Apical periodontitis
OP	Osteoporosis
PACS1	Phosphofurin acidic cluster sorting protein 1
DEGs	Differentially expressed genes
GO	Gene Ontology
KEGG	Kyoto Encyclopedia of Genes and Genomes
GSEA	Gene Set Enrichment Analysis
GSVA	Gene Set Variation Analysis
ssGSEA	single-sample gene set enrichment analysis
ROC	Receiver operating characteristic
AUC	Area under the curve
qRT-PCR	Quantitative real-time polymerase chain reaction
siRNA	Small interfering RNA
MHC class I	Major histocompatibility complex class I
iDCs	Immature dendritic cells
HLA	Human leukocyte antigen
CCR	Chemokine receptor
TILs	Tumor-infiltrating lymphocytes
TGN	Trans-Golgi network
IL-1β	Interleukin-1 beta
TNF-α	Tumor necrosis factor-alpha
IL-6	Interleukin-6
EMT	Epithelial-mesenchymal transition
