# Supplementary material for: Transcriptomic Analysis Reveals PACS1 as a Potential Shared Candidate Biomarker for Apical Periodontitis and Osteoporosis
Source: Int J Mol Sci. 2026 Jul 25;27(15):6646. doi: 10.3390/ijms27156646 (PMC13466771; doi:10.3390/ijms27156646)
Supplement: Supplementary file 1 [file ijms-27-06646-s001.zip › ijms-4358347-supplementary.pdf]

Supplementary Material

Transcriptomic Analysis Reveals PACS1 as a Potential Shared Candidate Biomarker for Apical Periodontitis and Osteoporosis

Saixuan Wu †, Sen Wang †, Huicong Bai, Jingyantong Zhang, Zhen Zhang, Ming Dong * and Weidong Niu *

School and Hospital of Stomatology, Dalian Medical University, Dalian 116044, China; wusx@dmu.edu.cn (S.W.); wangs29@dmu.edu.cn (S.W.); baihc@dmu.edu.cn (H.B.); zhangjingyantong@outlook.com (J.Z.); zhangz011210@163.com (Z.Z.)

* Correspondence: dongm@dmu.edu.cn (M.D.); niu.wd.endo@dmu.edu.cn (W.N.)

† These authors contributed equally to this work.

Study population and clinical examinations

This study was approved by the Ethics Committee of the Affiliated Stomatological Hospital of Dalian Medical University (approval number: 2025004), and all subjects signed written informed consent forms.

1 Inclusion Criteria

Control Group： Tissue source: Fully impacted third molars, or healthy periodontal ligaments from teeth with good periodontal status extracted for orthodontic treatment; Imaging findings: X-rays showed clear and continuous periapical membrane space, without widening or radiolucency. **Experimental Group：** Clinical and tissue source: Clinically confirmed chronic apical periodontitis The dental x-rays showed a periapical index (PAI) score≥3, affected teeth extracted therapeutically or periapical lesion tissues curetted during apical surgery; Bone mineral density requirement: BMD test T-score ≤ -2.5 (detection sites: lumbar spine, femoral neck or total hip).

2 Exclusion Criteria

Taking anti-inflammatory or antibiotic drugs within 3 months before tooth extraction or surgery; The affected tooth had periodontal disease (periodontal pocket depth > 4mm); Having secondary osteoporosis; Having other endocrine diseases affecting bone metabolism; Long-term use of drugs affecting bone metabolism; Having severe heart, lung, liver or kidney dysfunction; Being a malignant tumor patient.

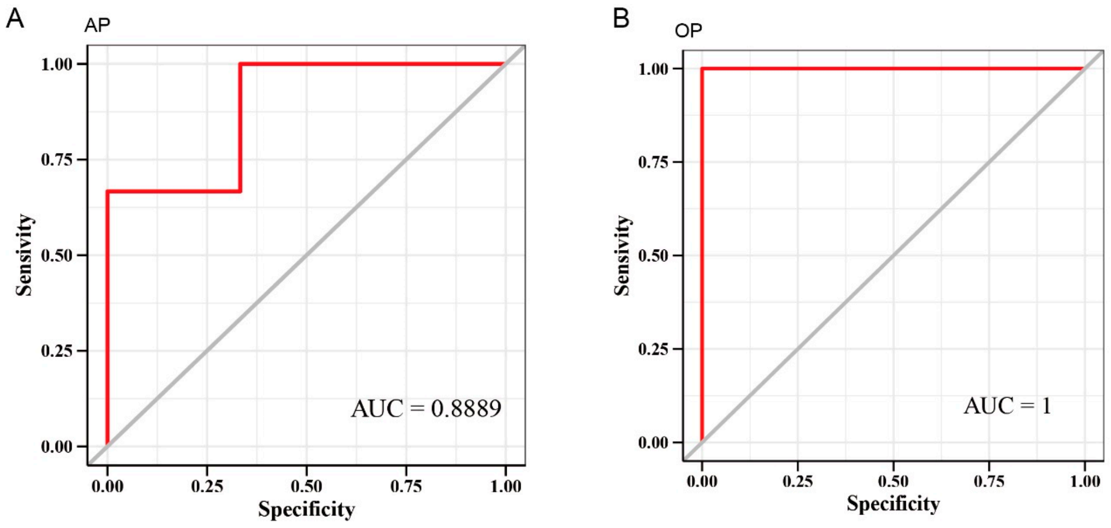

Figure S1. ROC curves of the random forest models for AP and OP. (A) ROC curve of the random forest model for AP. The AUC was 0.8889. (B) ROC curve of the random forest model for OP. The AUC was 1.0000.

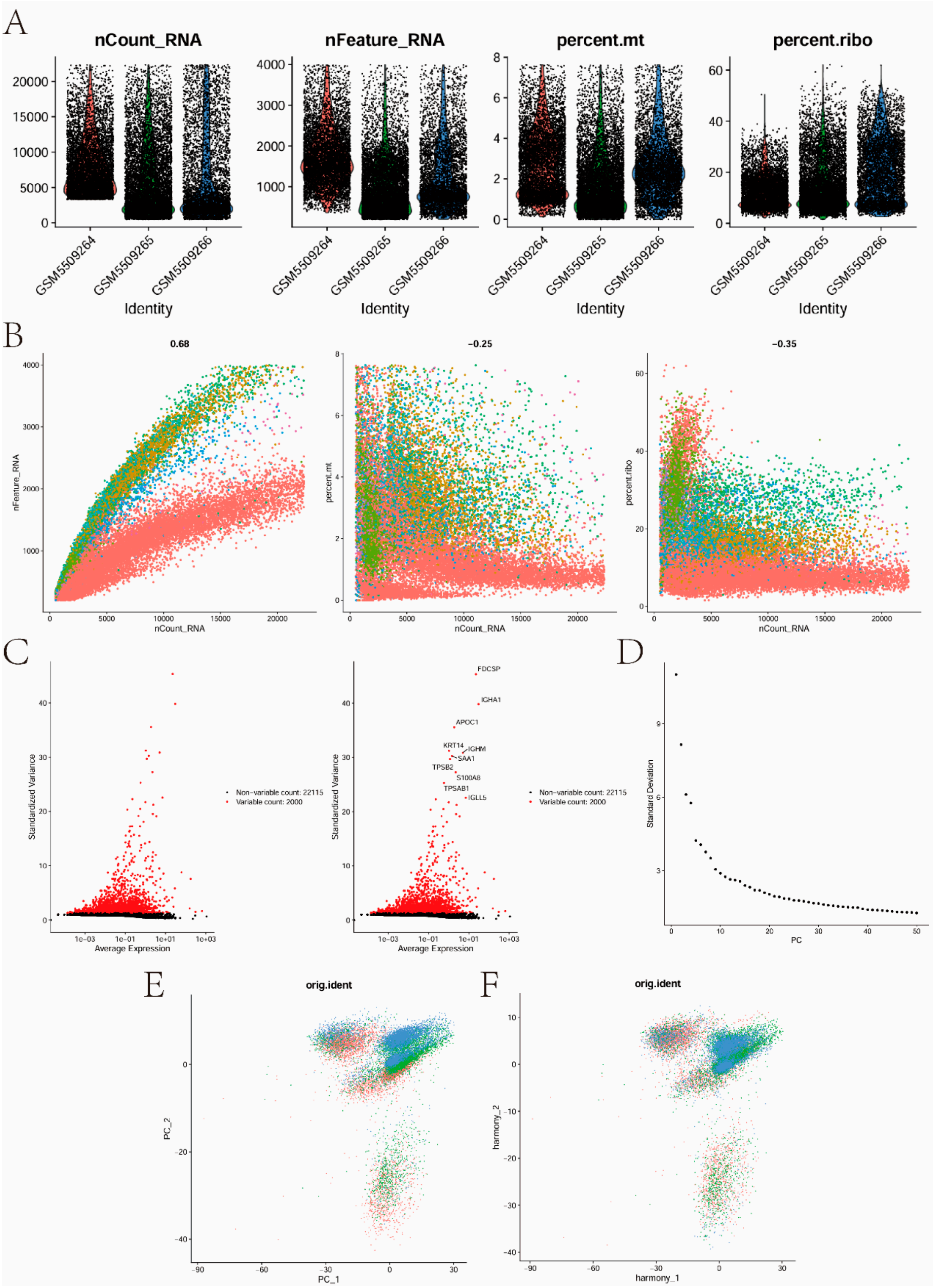

Figure S2. Quality control and clustering of AP scRNA-seq data. (A) Violin plots of key QC metrics of AP single cells after low-quality cell filtration and doublet removal. (B) Scatter plots of qualified AP cell distribution post-QC. (C) Identification of 2000 highly variable genes in AP scRNA-seq data by gene expression standard deviation. (D) PCA of standardized and homogenized AP single-cell transcriptome data. (E) Harmony batch effect correction analysis of AP single-cell PCA data.

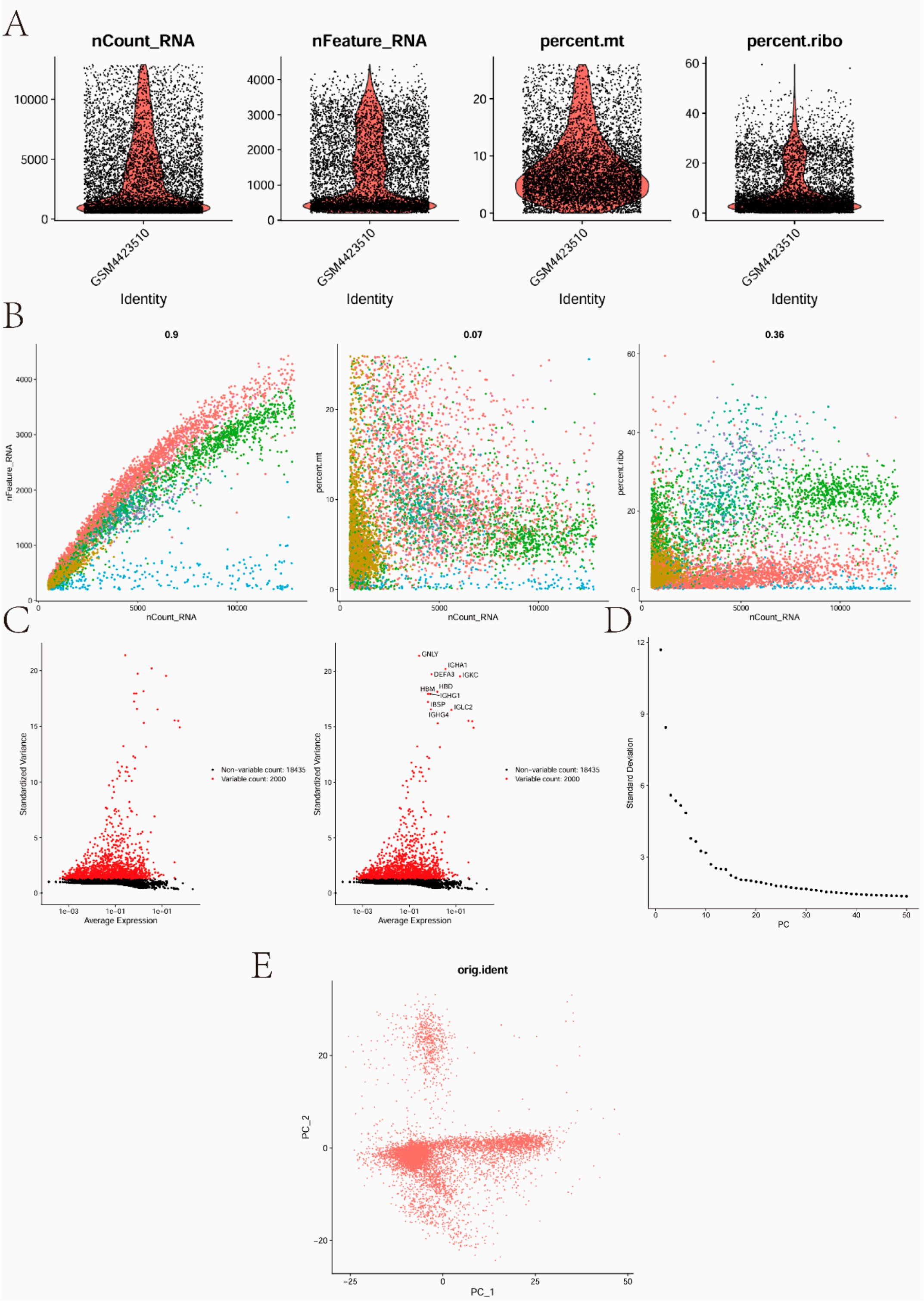

Figure S3. Quality control and clustering of OP scRNA-seq data. (A) Violin plots of key QC metrics of OP single cells after low-quality cell filtration and doublet removal. (B) Scatter plots of qualified OP cell distribution post-QC. (C) Identification of 2000 highly variable genes in OP scRNA-seq data by gene expression standard deviation. (D) PCA of standardized and homogenized OP single-cell transcriptome data. (E) Harmony batch effect correction analysis of OP single-cell PCA data.

Table S1. Demographic metadata of human populations with apical periodontitis

Cohort Group	Donors (n)	Gender	Age Range (years)	Race Distribution
Apical periodontitis	3	Male	62–89	Hispanic = 3
Apical periodontitis	3	Female	19–59	Hispanic = 2, White = 1
control	3	Male	22–82	Asian = 1, White = 2
control	3	Female	19–72	Hispanic = 2, White = 1

Table S2. Demographic metadata of human populations with osteoporosis

Cohort Group	Donors (n)	Gender	Average donor age (years)
control	5	4 Female, 1 Male	57.6 ± 9.56
Osteoporotic	5	5 Female	86.2 ± 5.89

Table S3. P values between key genes and metabolic pathways.

apical periodontitis				osteoporosis			
Symbol	pathway	cor	pvalue	Symbol	pathway	cor	pvalue
FAM87B	Cardiolipin Biosynthesis	-0.216783	0.498555985524189	FAM87B	Cardiolipin Biosynthesis	-0.212121	0.55630577510293
ASCL2	Cardiolipin Biosynthesis	-0.223776	0.484451661455399	ASCL2	Cardiolipin Biosynthesis	-0.478788	0.161522928017456
PACS1	Cardiolipin Biosynthesis	-0.34965	0.26523878689279	PACS1	Cardiolipin Biosynthesis	-0.272727	0.445838341542751
FAM87B	Cholesterol Biosynthesis	-0.258741	0.416774768899138	FAM87B	Cholesterol Biosynthesis	-0.551515	0.0984011766696349
ASCL2	Cholesterol Biosynthesis	0.013986013986014	0.965590268918779	ASCL2	Cholesterol Biosynthesis	-0.769697	0.00922195272221593

apical periodontitis				osteoporosis			
**Symbol**	**pathway**	**cor**	**pvalue**	**Symbol**	**pathway**	**cor**	**pvalue**
PACS1	Cholesterol Biosynthesis	-0.699301	0.011374198642691	PACS1	Cholesterol Biosynthesis	-0.575758	0.0815528147726023
FAM87B	Pyrimidine Biosynthesis	-0.741259	0.00580115047447816	FAM87B	Pyrimidine Biosynthesis	-0.393939	0.259997766834888
ASCL2	Pyrimidine Biosynthesis	-0.258741	0.416774768899138	ASCL2	Pyrimidine Biosynthesis	-0.478788	0.161522928017456
PACS1	Pyrimidine Biosynthesis	-0.797203	0.00190036772552826	PACS1	Pyrimidine Biosynthesis	-0.563636	0.0897240283170913
FAM87B	Dopamine Biosynthesis	-0.020979	0.948402225236522	FAM87B	Dopamine Biosynthesis	0.0424242424242424	0.907363817812816
ASCL2	Dopamine Biosynthesis	0.34965034965035	0.26523878689279	ASCL2	Dopamine Biosynthesis	-0.030303	0.933772958094147
PACS1	Dopamine Biosynthesis	0.0699300699300699	0.829023640531309	PACS1	Dopamine Biosynthesis	-0.345455	0.328226511471367
FAM87B	Epinephrine Biosynthesis	-0.034965	0.91409334241076	FAM87B	Epinephrine Biosynthesis	0.539393939393939	0.107593187782415
ASCL2	Epinephrine Biosynthesis	0.237762237762238	0.456800852639923	ASCL2	Epinephrine Biosynthesis	0.466666666666667	0.173938597165066
PACS1	Epinephrine Biosynthesis	0.118881118881119	0.712884219214772	PACS1	Epinephrine Biosynthesis	0.163636363636364	0.651477342796243
FAM87B	Norepinephrine Biosynthesis	-0.034965	0.91409334241076	FAM87B	Norepinephrine Biosynthesis	0.442424242424242	0.200422686711942
ASCL2	Norepinephrine Biosynthesis	0.237762237762238	0.456800852639923	ASCL2	Norepinephrine Biosynthesis	0.43030303030303	0.214492332532808
PACS1	Norepinephrine Biosynthesis	0.118881118881119	0.712884219214772	PACS1	Norepinephrine Biosynthesis	0.139393939393939	0.700931884910058
FAM87B	Fatty Acid Degradation	0.0629370629370629	0.845930921228779	FAM87B	Fatty Acid Degradation	-0.490909	0.149655673420837
ASCL2	Fatty Acid Degradation	0.398601398601399	0.199335494617809	ASCL2	Fatty Acid Degradation	-0.478788	0.161522928017456
PACS1	Fatty Acid Degradation	-0.328671	0.296904115688764	PACS1	Fatty Acid Degradation	-0.636364	0.0479117261299753
FAM87B	Transsulfuration	-0.517483	0.0848687711339349	FAM87B	Transsulfuration	-0.369697	0.293050075292822
ASCL2	Transsulfuration	-0.622378	0.0306758950616401	ASCL2	Transsulfuration	-0.078788	0.828717394697461
PACS1	Transsulfuration	-0.545455	0.0666118822668891	PACS1	Transsulfuration	0.381818181818182	0.276255333385436

apical periodontitis				osteoporosis			
**Symbol**	**pathway**	**cor**	**pvalue**	**Symbol**	**pathway**	**cor**	**pvalue**
FAM87B	Kynurenine Metabolism	0.776223776223776	0.00299286368018592	FAM87B	Kynurenine Metabolism	0.0909090909090909	0.802771731207162
ASCL2	Kynurenine Metabolism	0.321678321678322	0.307909876180653	ASCL2	Kynurenine Metabolism	-0.127273	0.726057014762789
PACS1	Kynurenine Metabolism	0.65034965034965	0.0220341909323654	PACS1	Kynurenine Metabolism	-0.454545	0.186904810349332
FAM87B	Retinoic Acid Metabolism	0.244755244755245	0.443261936450183	FAM87B	Retinoic Acid Metabolism	-0.733333	0.0158005962505716
ASCL2	Retinoic Acid Metabolism	0.433566433566434	0.159105769412196	ASCL2	Retinoic Acid Metabolism	-0.806061	0.00486206110196452
PACS1	Retinoic Acid Metabolism	0.153846153846154	0.633090681246262	PACS1	Retinoic Acid Metabolism	-0.757576	0.0111434467996943
FAM87B	Retinol Metabolism	0.244755244755245	0.443261936450183	FAM87B	Retinol Metabolism	0.527272727272727	0.117308065550202
ASCL2	Retinol Metabolism	0.503496503496504	0.0951573665941248	ASCL2	Retinol Metabolism	0.333333333333333	0.346593507087334
PACS1	Retinol Metabolism	-0.097902	0.762121655996299	PACS1	Retinol Metabolism	0.2	0.579584
FAM87B	Steroid Hormone Metabolism	0.0559440559440559	0.862898073694971	FAM87B	Steroid Hormone Metabolism	0.515151515151515	0.127552869703234
ASCL2	Steroid Hormone Metabolism	0.363636363636364	0.245265000730777	ASCL2	Steroid Hormone Metabolism	0.357575757575758	0.31037609170568
PACS1	Steroid Hormone Metabolism	-0.468531	0.124454791188834	PACS1	Steroid Hormone Metabolism	0.0545454545454545	0.881036181161853
FAM87B	Aldosterone Biosynthesis	0.0909090909090909	0.778725396245442	FAM87B	Aldosterone Biosynthesis	-0.10303	0.77699846344389
ASCL2	Aldosterone Biosynthesis	0.475524475524476	0.118175957013032	ASCL2	Aldosterone Biosynthesis	-0.006061	0.986742911194989
PACS1	Aldosterone Biosynthesis	-0.06993	0.829023640531309	PACS1	Aldosterone Biosynthesis	0.466666666666667	0.173938597165066
FAM87B	Cortisol Biosynthesis	0.223776223776224	0.484451661455399	FAM87B	Cortisol Biosynthesis	0.0787878787878788	0.828717394697461
ASCL2	Cortisol Biosynthesis	0.601398601398601	0.0385884529220193	ASCL2	Cortisol Biosynthesis	0.139393939393939	0.700931884910058
PACS1	Cortisol Biosynthesis	0.0489510489510490	0.879918578878675	PACS1	Cortisol Biosynthesis	0.539393939393939	0.107593187782415
FAM87B	Estradiol Biosynthesis	0.342657342657343	0.27556745095678	FAM87B	Estradiol Biosynthesis	-0.212121	0.55630577510293

apical periodontitis				osteoporosis			
**Symbol**	**pathway**	**cor**	**pvalue**	**Symbol**	**pathway**	**cor**	**pvalue**
ASCL2	Estradiol Biosynthesis	0.685314685314685	0.0139059688143628	ASCL2	Estradiol Biosynthesis	-0.054545	0.881036181161853
PACS1	Estradiol Biosynthesis	0.237762237762238	0.456800852639923	PACS1	Estradiol Biosynthesis	0.236363636363636	0.5108853175152
FAM87B	Testosterone Biosynthesis	0.503496503496504	0.0951573665941248	FAM87B	Testosterone Biosynthesis	-0.115152	0.751419652325848
ASCL2	Testosterone Biosynthesis	0.755244755244755	0.00450843548429436	ASCL2	Testosterone Biosynthesis	-0.115152	0.751419652325848
PACS1	Testosterone Biosynthesis	0.272727272727273	0.391096770941896	PACS1	Testosterone Biosynthesis	0.236363636363636	0.5108853175152
FAM87B	Urea Cycle	-0.265734	0.403833088314712	FAM87B	Urea Cycle	0.0666666666666667	0.854813088248743
ASCL2	Urea Cycle	0.0769230769230769	0.812182698028059	ASCL2	Urea Cycle	-0.018182	0.960240418128624
PACS1	Urea Cycle	-0.657343	0.020185498509854	PACS1	Urea Cycle	0.36969696969697	0.293050075292822
FAM87B	Vitamin K	-0.692308	0.0125930304960939	FAM87B	Vitamin K	0.333333333333333	0.346593507087334
ASCL2	Vitamin K	-0.391608	0.208063463695353	ASCL2	Vitamin K	0.406060606060606	0.244282294086626
PACS1	Vitamin K	-0.734266	0.00654349014683797	PACS1	Vitamin K	0.139393939393939	0.700931884910058
FAM87B	Pentose and Glucuronate Interconversions	-0.160839	0.617523489540142	FAM87B	Pentose and Glucuronate Interconversions	-0.163636	0.651477342796243
ASCL2	Pentose and Glucuronate Interconversions	0.111888111888112	0.729194990751066	ASCL2	Pentose and Glucuronate Interconversions	-0.260606	0.46708905438634
PACS1	Pentose and Glucuronate Interconversions	-0.384615	0.217020225759977	PACS1	Pentose and Glucuronate Interconversions	-0.090909	0.802771731207162
FAM87B	Ascorbate and Aldrate Metabolism	-0.132867	0.680597508286082	FAM87B	Ascorbate and Aldrate Metabolism	-0.151515	0.676065175997854
ASCL2	Ascorbate and Aldrate Metabolism	0.118881118881119	0.712884219214772	ASCL2	Ascorbate and Aldrate Metabolism	-0.10303	0.77699846344389
PACS1	Ascorbate and Aldrate Metabolism	-0.342657	0.27556745095678	PACS1	Ascorbate and Aldrate Metabolism	0.0303030303030303	0.933772958094147

apical periodontitis				osteoporosis			
Symbol	pathway	cor	pvalue	Symbol	pathway	cor	pvalue
FAM87B	Starch and Suctose Metabolism	0.384615384615385	0.217020225759977	FAM87B	Starch and Suctose Metabolism	0.381818181818182	0.276255333385436
ASCL2	Starch and Suctose Metabolism	0.566433566433566	0.0548421197754076	ASCL2	Starch and Suctose Metabolism	0.248484848484848	0.488776304519243
PACS1	Starch and Suctose Metabolism	0.223776223776224	0.484451661455399	PACS1	Starch and Suctose Metabolism	0.442424242424242	0.200422686711942
FAM87B	Glyoxylate and Dicarboxylate Metabolism	-0.034965	0.91409334241076	FAM87B	Glyoxylate and Dicarboxylate Metabolism	-0.006061	0.986742911194989
ASCL2	Glyoxylate and Dicarboxylate Metabolism	0.342657342657343	0.27556745095678	ASCL2	Glyoxylate and Dicarboxylate Metabolism	-0.127273	0.726057014762789
PACS1	Glyoxylate and Dicarboxylate Metabolism	-0.454545	0.13765791634773	PACS1	Glyoxylate and Dicarboxylate Metabolism	-0.018182	0.960240418128624
FAM87B	Propanoate Metabolism	-0.188811	0.556737056176831	FAM87B	Propanoate Metabolism	-0.672727	0.0330412225454377
ASCL2	Propanoate Metabolism	0.0839160839160839	0.795414501853898	ASCL2	Propanoate Metabolism	-0.587879	0.073877706888658
PACS1	Propanoate Metabolism	-0.615385	0.0331699010901059	PACS1	Propanoate Metabolism	-0.709091	0.0216659233675323
FAM87B	Butanoate Metabolism	0.174825174825175	0.586823664362705	FAM87B	Butanoate Metabolism	-0.672727	0.0330412225454377
ASCL2	Butanoate Metabolism	0.440559440559441	0.15173503571673	ASCL2	Butanoate Metabolism	-0.769697	0.00922195272221593
PACS1	Butanoate Metabolism	-0.223776	0.484451661455399	PACS1	Butanoate Metabolism	-0.915152	0.000204472406148959
FAM87B	Primary Bile Acid Biosynthesis	0.111888111888112	0.729194990751066	FAM87B	Primary Bile Acid Biosynthesis	0.0303030303030303	0.933772958094147
ASCL2	Primary Bile Acid Biosynthesis	0.104895104895105	0.745609480980708	ASCL2	Primary Bile Acid Biosynthesis	0.248484848484848	0.488776304519243
PACS1	Primary Bile Acid Biosynthesis	0.258741258741259	0.416774768899138	PACS1	Primary Bile Acid Biosynthesis	-0.175758	0.627188344776485

apical periodontitis				osteoporosis			
**Symbol**	**pathway**	**cor**	**pvalue**	**Symbol**	**pathway**	**cor**	**pvalue**
FAM87B	Steroid Hormone Biosynthesis	-0.06993	0.829023640531309	FAM87B	Steroid Hormone Biosynthesis	0.442424242424242	0.200422686711942
ASCL2	Steroid Hormone Biosynthesis	0.034965034965035	0.91409334241076	ASCL2	Steroid Hormone Biosynthesis	0.236363636363636	0.5108853175152
PACS1	Steroid Hormone Biosynthesis	-0.531469	0.0753623350534838	PACS1	Steroid Hormone Biosynthesis	-0.127273	0.726057014762789
FAM87B	Linoleic Acid Metabolism	0.475524475524476	0.118175957013032	FAM87B	Linoleic Acid Metabolism	0.357575757575758	0.31037609170568
ASCL2	Linoleic Acid Metabolism	0.475524475524476	0.118175957013032	ASCL2	Linoleic Acid Metabolism	0.503030303030303	0.138333698394492
PACS1	Linoleic Acid Metabolism	0.391608391608392	0.208063463695353	PACS1	Linoleic Acid Metabolism	0.515151515151515	0.127552869703234
FAM87B	Glycine, Serine and Threonine Metabolism	-0.034965	0.91409334241076	FAM87B	Glycine, Serine and Threonine Metabolism	0.575757575757576	0.0815528147726023
ASCL2	Glycine, Serine and Threonine Metabolism	0.335664335664336	0.286122902914987	ASCL2	Glycine, Serine and Threonine Metabolism	0.696969696969697	0.0250966758822517
PACS1	Glycine, Serine and Threonine Metabolism	-0.398601	0.199335494617809	PACS1	Glycine, Serine and Threonine Metabolism	0.76969696969697	0.00922195272221593
FAM87B	Valine, Leucine and Isoleucine Degradation	-0.027972	0.931234351201881	FAM87B	Valine, Leucine and Isoleucine Degradation	-0.733333	0.0158005962505716
ASCL2	Valine, Leucine and Isoleucine Degradation	0.314685314685315	0.319138785503305	ASCL2	Valine, Leucine and Isoleucine Degradation	-0.69697	0.0250966758822517
PACS1	Valine, Leucine and Isoleucine Degradation	-0.461538	0.130948069507173	PACS1	Valine, Leucine and Isoleucine Degradation	-0.769697	0.00922195272221593
FAM87B	Valine, Leucine and Isoleucine Biosynthesis	0.566433566433566	0.0548421197754076	FAM87B	Valine, Leucine and Isoleucine Biosynthesis	-0.054545	0.881036181161853
ASCL2	Valine, Leucine and	0.748251748251748	0.005124081698753	ASCL2	Valine, Leucine and	-0.030303	0.933772958094147

apical periodontitis				osteoporosis			
Symbol	pathway	cor	pvalue	Symbol	pathway	cor	pvalue
	Isoleucine Biosynthesis				Isoleucine Biosynthesis		
PACS1	Valine, Leucine and Isoleucine Biosynthesis	0.58041958041958	0.0478559768420606	PACS1	Valine, Leucine and Isoleucine Biosynthesis	0.272727272727273	0.445838341542751
FAM87B	Arginine Biosynthesis	-0.188811	0.556737056176831	FAM87B	Arginine Biosynthesis	0.115151515151515	0.751419652325848
ASCL2	Arginine Biosynthesis	0.146853146853147	0.648795709784666	ASCL2	Arginine Biosynthesis	0.0424242424242424	0.907363817812816
PACS1	Arginine Biosynthesis	-0.398601	0.199335494617809	PACS1	Arginine Biosynthesis	0.296969696969697	0.404701671270157
FAM87B	Histidine Metabolism	0.20979020979021	0.512840943181826	FAM87B	Histidine Metabolism	-0.151515	0.676065175997854
ASCL2	Histidine Metabolism	0.426573426573427	0.166699987624945	ASCL2	Histidine Metabolism	0.0787878787878788	0.828717394697461
PACS1	Histidine Metabolism	0.0979020979020979	0.762121655996299	PACS1	Histidine Metabolism	-0.393939	0.259997766834888
FAM87B	Tyrosine Metabolism	0.503496503496504	0.0951573665941248	FAM87B	Tyrosine Metabolism	0.260606060606061	0.46708905438634
ASCL2	Tyrosine Metabolism	0.713286713286713	0.00920177977763492	ASCL2	Tyrosine Metabolism	0.454545454545455	0.186904810349332
PACS1	Tyrosine Metabolism	0.265734265734266	0.403833088314712	PACS1	Tyrosine Metabolism	0.151515151515152	0.676065175997854
FAM87B	Phenylalanine Metabolism	0.496503496503497	0.100602631832255	FAM87B	Phenylalanine Metabolism	0.103030303030303	0.77699846344389
ASCL2	Phenylalanine Metabolism	0.671328671328671	0.0168314561868934	ASCL2	Phenylalanine Metabolism	0.284848484848485	0.425038154892146
PACS1	Phenylalanine Metabolism	0.216783216783217	0.498555985524189	PACS1	Phenylalanine Metabolism	-0.054545	0.881036181161853
FAM87B	Tryptophan Metabolism	0.755244755244755	0.00450843548429436	FAM87B	Tryptophan Metabolism	-0.284848	0.425038154892146
ASCL2	Tryptophan Metabolism	0.713286713286713	0.00920177977763492	ASCL2	Tryptophan Metabolism	-0.321212	0.36546831043867
PACS1	Tryptophan Metabolism	0.538461538461538	0.0708943175492232	PACS1	Tryptophan Metabolism	-0.69697	0.0250966758822517
FAM87B	Phenylalanine, Tyrosine and Tryptophan Biosynthesis	0.153846153846154	0.633090681246262	FAM87B	Phenylalanine, Tyrosine and Tryptophan Biosynthesis	0.36969696969697	0.293050075292822

apical periodontitis				osteoporosis			
**Symbol**	**pathway**	**cor**	**pvalue**	**Symbol**	**pathway**	**cor**	**pvalue**
ASCL2	Phenylalanine, Tyrosine and Tryptophan Biosynthesis	0.244755244755245	0.443261936450183	ASCL2	Phenylalanine, Tyrosine and Tryptophan Biosynthesis	0.0787878787878788	0.828717394697461
PACS1	Phenylalanine, Tyrosine and Tryptophan Biosynthesis	-0.174825	0.586823664362705	PACS1	Phenylalanine, Tyrosine and Tryptophan Biosynthesis	0.345454545454545	0.328226511471367
FAM87B	Beta-Alanine Metabolism	0.517482517482518	0.0848687711339349	FAM87B	Beta-Alanine Metabolism	-0.357576	0.31037609170568
ASCL2	Beta-Alanine Metabolism	0.629370629370629	0.0283196712957601	ASCL2	Beta-Alanine Metabolism	-0.248485	0.488776304519243
PACS1	Beta-Alanine Metabolism	0.391608391608392	0.208063463695353	PACS1	Beta-Alanine Metabolism	-0.454545	0.186904810349332
FAM87B	Biotin Metabolism	-0.335664	0.286122902914987	FAM87B	Biotin Metabolism	0.212121212121212	0.55630577510293
ASCL2	Biotin Metabolism	-0.370629	0.235621003512646	ASCL2	Biotin Metabolism	-0.10303	0.77699846344389
PACS1	Biotin Metabolism	-0.608392	0.0358059970520292	PACS1	Biotin Metabolism	-0.054545	0.881036181161853
FAM87B	Lipoic Acid Metabolism	0.293706293706294	0.354147641232922	FAM87B	Lipoic Acid Metabolism	-0.321212	0.36546831043867
ASCL2	Lipoic Acid Metabolism	0.251748251748252	0.429918785032884	ASCL2	Lipoic Acid Metabolism	-0.454545	0.186904810349332
PACS1	Lipoic Acid Metabolism	0.0839160839160839	0.795414501853898	PACS1	Lipoic Acid Metabolism	-0.151515	0.676065175997854
FAM87B	Metabolism of Xenobiotics by Cytochrome P450	0.412587412587413	0.182563978573167	FAM87B	Metabolism of Xenobiotics by Cytochrome P450	0.224242424242424	0.533400561272595
ASCL2	Metabolism of Xenobiotics by Cytochrome P450	0.657342657342657	0.0201854985098854	ASCL2	Metabolism of Xenobiotics by Cytochrome P450	0.466666666666667	0.173938597165066
PACS1	Metabolism of Xenobiotics by	0.0559440559440559	0.862898073694971	PACS1	Metabolism of Xenobiotics by	0.357575757575758	0.31037609170568

apical periodontitis				osteoporosis			
Symbol	pathway	cor	pvalue	Symbol	pathway	cor	pvalue
	Cytochrome P450				Cytochrome P450		
FAM87B	Drug Metabolism by Cytochrome P450	0.0839160839160839	0.795414501853898	FAM87B	Drug Metabolism by Cytochrome P450	0.163636363636364	0.651477342796243
ASCL2	Drug Metabolism by Cytochrome P450	0.377622377622378	0.226206044935027	ASCL2	Drug Metabolism by Cytochrome P450	0.575757575757576	0.0815528147726023
PACS1	Drug Metabolism by Cytochrome P450	-0.041958	0.896985870819554	PACS1	Drug Metabolism by Cytochrome P450	0.381818181818182	0.276255333385436
FAM87B	Drug Metabolism by other enzymes	0.503496503496504	0.0951573665941248	FAM87B	Drug Metabolism by other enzymes	0.442424242424242	0.200422686711942
ASCL2	Drug Metabolism by other enzymes	0.685314685314685	0.0139059688143628	ASCL2	Drug Metabolism by other enzymes	0.478787878787879	0.161522928017456
PACS1	Drug Metabolism by other enzymes	0.286713286713287	0.366251460583582	PACS1	Drug Metabolism by other enzymes	0.660606060606061	0.0375883775714092
